# Novel application of cryobiopsy in the diagnosis of pulmonary alveolar proteinosis

**DOI:** 10.1002/rcr2.336

**Published:** 2018-06-20

**Authors:** Meng‐fang Shen, Teressa Reanne Ju, Chi Chan Lee, Chih‐Yen Tu

**Affiliations:** ^1^ School of Medicine China Medical University Taichung Taiwan; ^2^ Division of Pulmonology, Department of Internal Medicine China Medical University Hospital Taichung Taiwan; ^3^ Mackay Memorial Hospital Taipei Taiwan; ^4^ Oregon Health & Science University Hospital Portland OR USA

**Keywords:** Cryobiopsy, forceps biopsy, pulmonary alveolar proteinosis

## Abstract

Pulmonary alveolar proteinosis (PAP) is a rare disease diagnosed pathologically by the build up of surfactant in the alveolar spaces. Establishing a diagnosis usually requires invasive procedures such as bronchoalveolar lavage and forceps biopsy to obtain tissue specimens. Infrequently, surgery is required when histopathological results from other modalities are equivocal. Cryobiopsy has emerged as a novel technique for obtaining lung tissues in pulmonary diseases. Recently, cryobiopsy has been used to diagnose diffuse parenchymal lung disease, but it has rarely been used for the diagnosis of PAP. Here, we describe a 54‐year‐old male businessman presenting with intermittent coughing with yellowish sputum and dyspnoea upon exertion for half a year. Tissues from forceps biopsy fail to yield a specific diagnosis, whereas those from cryobiopsy confirm the diagnosis of PAP. Cryobiopsy offers several diagnostic advantages compared to conventional techniques and appears to be a potential diagnostic tool for diagnosing PAP.

## Introduction

Pulmonary alveolar proteinosis (PAP) is a rare disease diagnosed pathologically by the build up of surfactant in the alveolar spaces. Symptoms and physical examinations are mostly non‐specific. Typical findings were obtained on chest computed tomography (CT). Images may show ground‐glass opacities superimposed on septal thickening, described as a “crazy paving pattern” 1. The diagnosis is traditionally established by pathological proof of tissue, either through bronchoscopy or video‐assisted thoracoscopic surgery (VATS). Cryobiopsy has emerged as a new modality to obtain histopathological specimens 2, 3. Here, we present a case of a 54‐year‐old man with a chief complaint of progressive shortness of breath. Conventional forceps biopsy and cryobiopsy were both performed, and only cryobiopsy revealed the pathological findings suggesting PAP.

## Case Report

A 54‐year‐old male businessman presented to our clinic with progressive shortness of breath for the last three months. He also complained of intermittent cough with yellowish sputum and dyspnoea upon exertion for half a year. He had a history of well‐controlled hypertension and smoking one pack of cigarettes per day. Family history was remarkable for adenocarcinoma of the lung. Travel history was remarkable for travel to Yunnan, China, in the past year.

**Figure 1:**
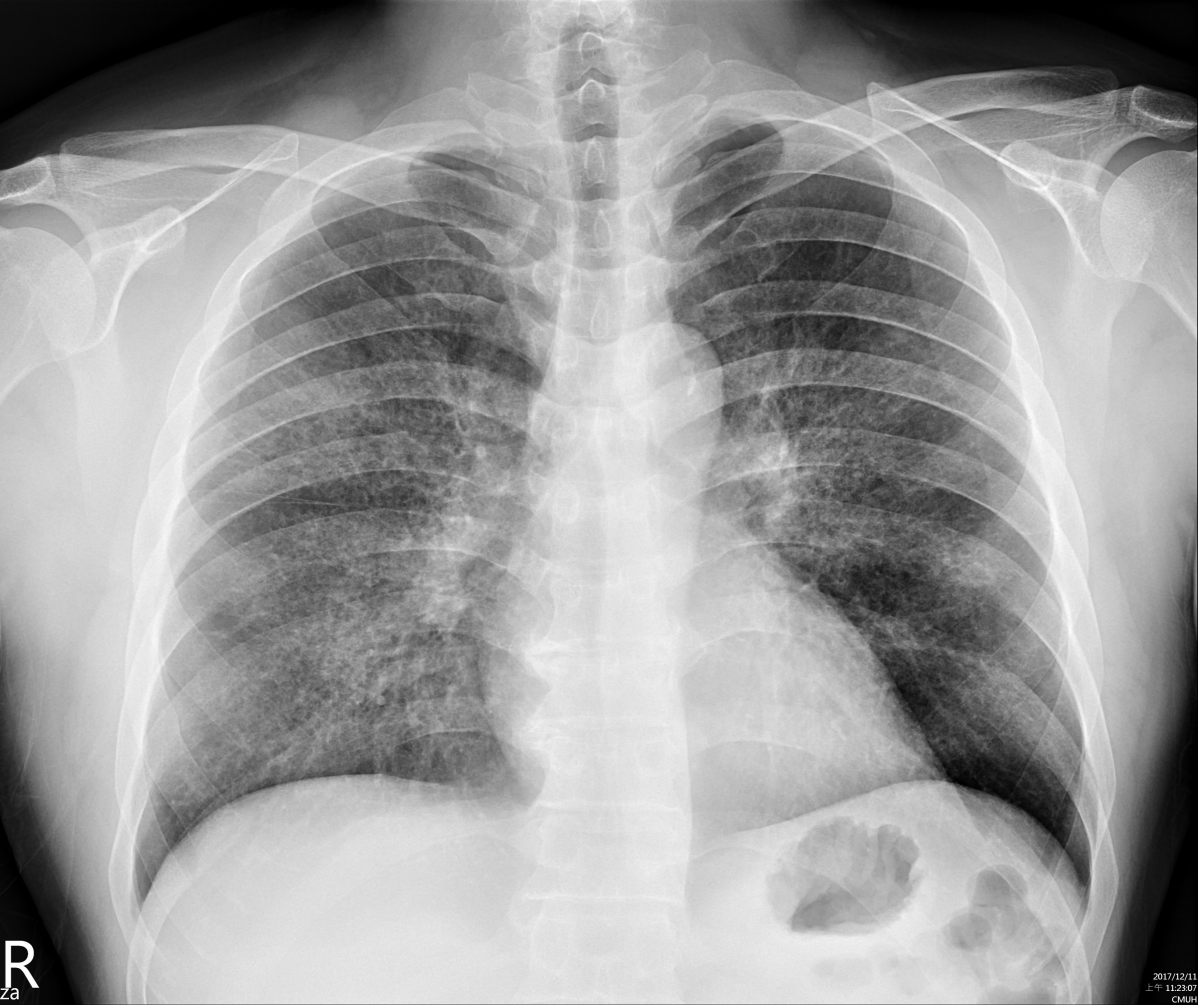
Chest X‐ray revealed non‐specific interstitial infiltration over bilateral lung fields.

He first visited a pulmonologist in a local clinic a month prior to admission, where chest X‐ray demonstrated increased infiltration over bilateral lung fields. Oral azithromycin was administered, but his dyspnoea progressed. He then visited our clinic for a second opinion. Upon examination, he denied fever, joint pain, dry eyes or mouth, muscle weakness, pitting oedema over lower legs, and skin rash. Physical examination was unremarkable, except chest auscultation demonstrating fine crackles over bilateral lung fields. Chest X‐ray showed diffuse interstitial infiltration bilaterally (Fig. 1). He was admitted for further workup and monitoring of his respiratory performance.

**Figure 2:**
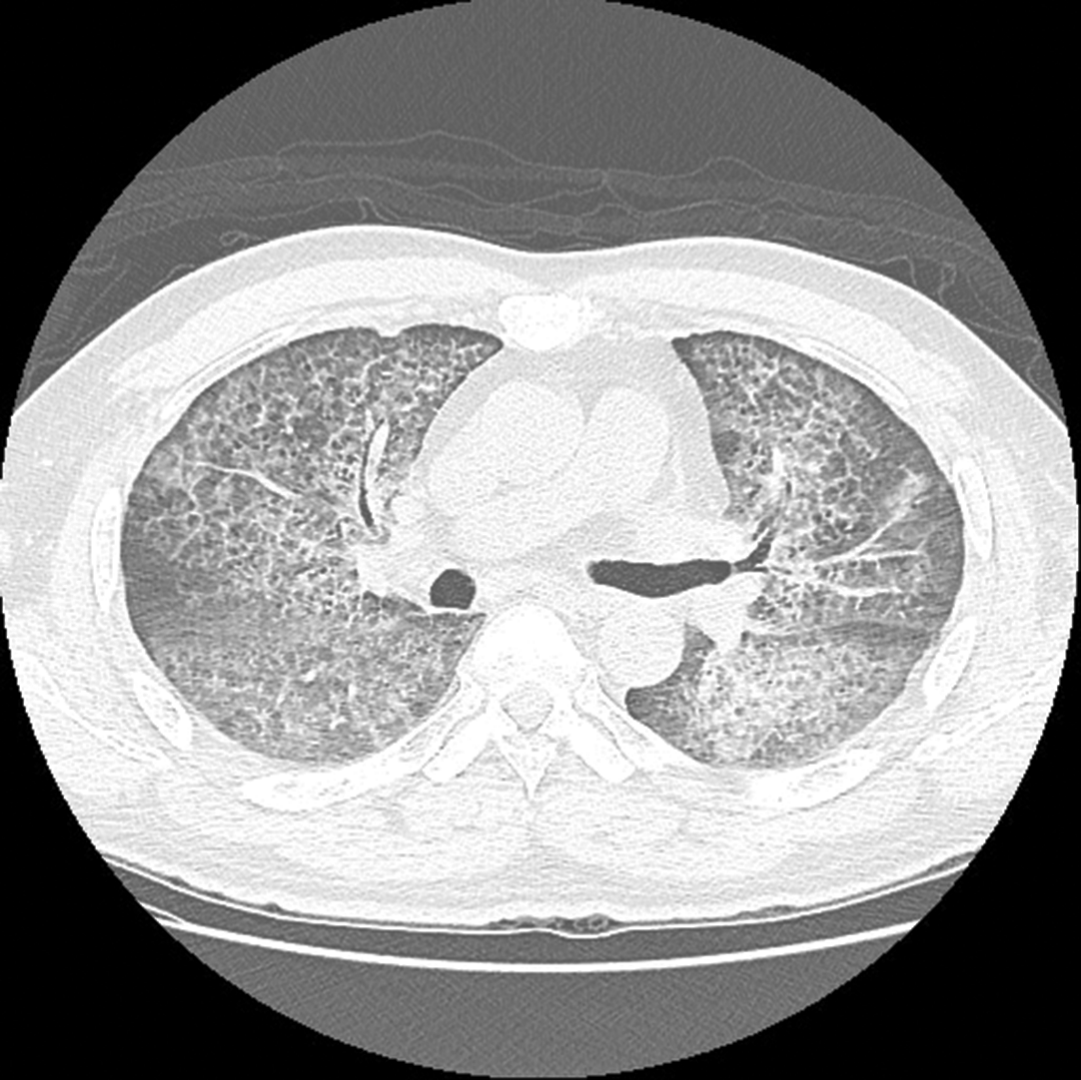
On chest computed tomography (CT), extensive patchy ground‐glass opacities superimposed with thickened interlobular septa and intralobular lines were noted in bilateral lungs, indicating a “crazy paving” pattern.

During hospitalization, the patient had no fever, and respiratory pattern was smooth. Peripheral capillary oxygen saturation (SpO_2_) was 95% under ambient air. Serum white blood cell count showed no leucocytosis or left shift. Autoimmune markers, including rheumatoid factor, antinuclear antibody, anti‐Smith antibody, anti‐ribonucleoprotein antibody, anti‐SSA, anti‐SSB, and anti‐Scl‐70, were all negative. Polymerase chain reaction of Pneumocystis jiroveci from induced sputum was negative. One of three sets of sputum culture demonstrated non‐tuberculous mycobacteria, which was later attributed to environmental contamination given his symptoms and image findings. Chest CT disclosed extensive patchy ground‐glass opacities superimposed with thickened interlobular septa and intralobular lines over both lung fields, demonstrating a “crazy paving” pattern (Fig.2). Pulmonary function test showed normal spirometry and moderately reduced diffusing capacity of the lungs for carbon monoxide (DLCO) on single breath diffusing capacity test (5.42 mmol/min/kPa, correlated with 57.7% of predicted value).

**Figure 3:**
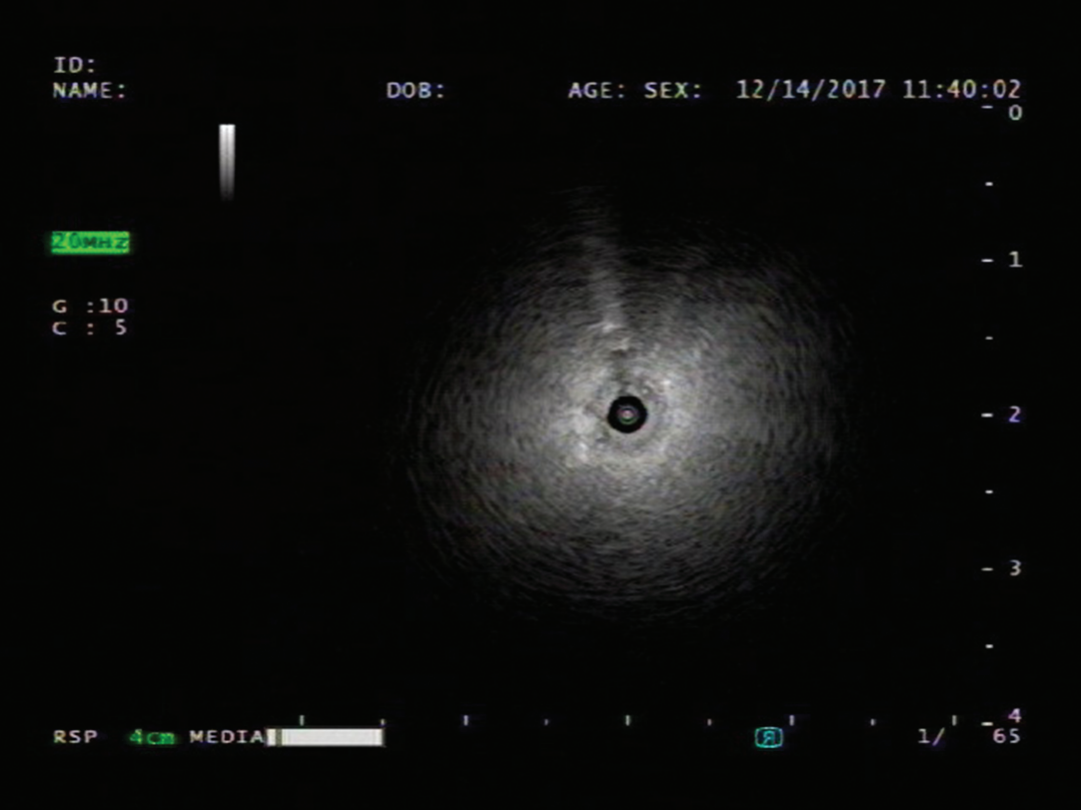
Radial probe endobronchial ultrasound (EBUS) showed a “blizzard sign”, suggesting ground‐glass opacity over the superior segment of right lower lobe.

Bronchoscopy indicated no endobronchial lesions. A radial probe endobronchial ultrasound (EBUS) showed a “blizzard sign”, suggesting ground‐glass opacity over the superior segment of the right lower lobe (Fig.3) 4. Brush cytology, bronchoalveolar lavage (BAL), and biopsy were performed. Brush cytology was negative for malignant cells.

Macroscopically, the BAL specimen was clear in appearance. Microbiological workup of BAL was negative for presence of bacteria, tubercolosis (TB), non‐tuberculous mycobacteria (NTM), fungus, and Pneumocystis jiroveci. We performed both a forceps biopsy and a cryobiopsy on the larger‐sized lesions.

Pathological report of the four 0.2 × 0.2 × 0.1 cm tissue fragments obtained by forceps biopsy indicated only chronic inflammation.

**Figure 4:**
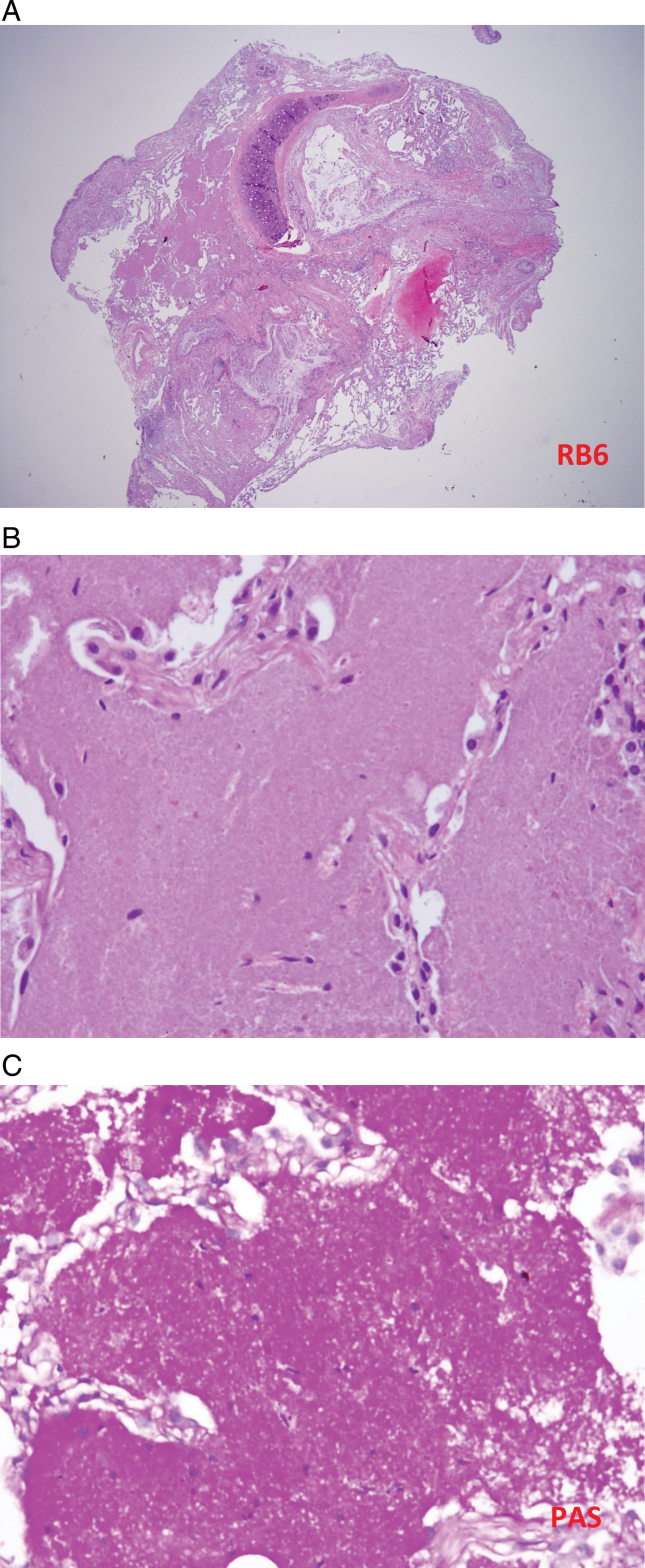
(A) Specimen of cryobiopsy contains intact architecture of tissues, including cartilage, alveoli, and interstitial space. Circled is eosinophilic granular material within the alveolar space. (B) Under high magnification, there is granular proteinaceous exudate filling the alveolar space and mild interstitial lymphocytic infiltration. (C) Intra‐alveolar material is strongly reactive to periodic acid‐Schiff staining.

On the other hand, the four 0.5 × 0.5 × 0.2 cm tissue fragments acquired by cryobiopsy revealed periodic acid‐Schiff (PAS)‐positive granular proteinaceous exudate with mild interstitial lymphocytic infiltration filling the alveolar spaces consistent with PAP (Fig.4).

We discussed the treatment options with the patient, and whole lung lavage was advised. The patient declined our suggested treatment and opted for close surveillance. He was discharged with outpatient follow up. Later, his shortness of breath spontaneously resolved, and serial chest X‐rays showed resolution of the interstitial infiltration of the lungs.

## Discussion

Pulmonary alveolar proteinosis was first reported by Rosen et al. in 1958 as a disease in which proteinaceous material fills the alveoli 1. Initial presentation of the disease is usually non‐specific, such as shortness of breath or dyspnoea on exertion. If cough is present, it is often mild with scant white or “chunky” sputum 2, 3, 4. Physical examination findings include inspiratory crackles and cyanosis 2. Less commonly, fever, chest pain, or haemoptysis also occurs, especially if secondary infection is present. In our case, the patient also had non‐specific symptoms, such as progressive shortness of breath and yellow sputum.

The aetiology of PAP can be divided into congenital, secondary, and autoimmune 2, where the end result is the build up of surfactant in alveolar spaces from the lack of clearance by macrophage. Supportive care is the mainstay of treatment for congenital PAP, whereas treatment of the underlying disease is the management for secondary PAP. For autoimmune PAP, whole lung lavage is the mainstay of therapy; granulocyte macrophage‐colony stimulating factor (GM‐CSF) therapy may be considered an alternative to whole lung lavage 5.

The diagnosis of PAP generally requires radiological imaging and histopathology specimens. Chest CT findings are notable for reticulations superimposed on ground‐glass opacities, also known as “crazy paving” patterns 2, 5. Recently, BAL has been used increasingly to diagnose PAP. The fluid from BAL is typically filled with eosinophilic, granular, PAS‐positive material and foamy alveolar macrophages 6. Trapnell et al. noted that, in about 75% of suspected cases, tissue sampling from BAL could adequately establish the diagnosis 2. Traditional forceps biopsy alone yielded a diagnostic rate of 73.1%, and multiple specimens were needed to establish the diagnosis 7. Combination of BAL, brush cytology, and forceps biopsy improved diagnostic sensitivity to 85.3% 3, yet it inevitably prolonged procedure time and increased cost. Rarely, VATS was required to obtain biopsy specimens. While VATS carries a sensitivity and specificity of 90% in diagnosing PAP, the incidence of pneumothorax and severe bleeding were significantly higher than other modalities 4.

Flexible cryoprobes were first used to diagnose and manage central airway lesions 8, 9, 10. In recent years, transbronchial cryobiopsy had been applied to the diagnosis of parenchymal lung disease 4. Cryobiopsy has the advantage of keeping the tissue architecture intact while being able to acquire an adequately sized specimen size compared to forceps biopsy 8, 9. Moreover, cryobiopsy has a lower risk of developing the associated complications and a shorter length of hospital stay compared to surgical biopsy 5.

In our case, we performed both forceps biopsy and cryobiopsy for the lung lesions. Diagnosis of PAP was made from specimens obtained from cryobiopsy instead of those from forceps biopsy. This discrepancy can be explained by a number of reasons. Firstly, the size of the cryobiopsy specimens was 0.5 × 0.5 × 0.2 cm, almost twice the size of the forceps biopsy specimens. A larger tissue sample may allow greater analysis of the lesion. Secondly, approaching an endobronchial lesion may be easier with cryoprobes than with forceps. Poletti et al. noted that the approach to a lesion with the forceps biopsy tool is parallel to the bronchus. As a result, a lesion may be more difficult to approach with forceps than with cryoprobes, which allows tissue sampling in the lateral direction 8. Finally, cryobiopsy allows preservation of the tissue architecture and possibly leads to greater diagnostic accuracy.

Diagnosis of PAP through cryobiopsy has been rarely reported in previous literature. Poletti et al. 8 published a case series that included 176 patients with diffuse parenchymal lung diseases (DPLD) who received cryobiopsy. Among them, one patient was diagnosed with PAP through cryobiopsy. Ussavarungsi et al. retrospectively analysed 74 cases with DPLD by cryobiopsy, and one patient from their study was diagnosed with PAP 10. Gando et al. also reported a case of PAP that was diagnosed by cryobiopsy 9. However, conventional forceps biopsy was not performed in their case. While a small number of cases were reported, cryobiopsy appears to be an excellent tool, with increasing potential for diagnosing PAP and other DPLD.

In conclusion, we present the second case report in the literature in which cryobiopsy was used to diagnosis PAP. Cryobiopsy potentially increases diagnostic accuracy compared to conventional techniques and carries a lower risk of procedural complications. Performing cryobiopsy to obtain specimens should be considered while DPLP is present and PAP is suspected. Further studies are required to elucidate the role of cryobiopsy in diagnosing PAP.

### Disclosure Statement

Appropriate written informed consent was obtained for publication of this case report and accompanying images.
